# Sound Localization and Lateralization by Bilateral Bone Conduction Devices, Middle Ear Implants, and Cartilage Conduction Hearing Aids

**DOI:** 10.3390/audiolres11040046

**Published:** 2021-09-30

**Authors:** Kimio Shiraishi

**Affiliations:** Department of Communication Design Science, Faculty of Design, Kyushu University, Fukuoka 815-0032, Japan; shiraishi.kimio.356@m.kyushu-u.ac.jp; Tel.: +81-92-804-8657

**Keywords:** localization, lateralization, binaural hearing, hearing loss, bone conduction device, middle ear implant, cartilage conduction hearing aid

## Abstract

Sound localization in daily life is one of the important functions of binaural hearing. Bilateral bone conduction devices (BCDs), middle ear implants, and cartilage conduction hearing aids have been often applied for patients with conductive hearing loss (CHL) or mixed hearing loss, for example, resulting from bilateral microtia and aural atresia. In this review, factors affecting the accuracy of sound localization with bilateral BCDs, middle ear implants, and cartilage conduction hearing aids were classified into four categories: (1) types of device, (2) experimental conditions, (3) participants, and (4) pathways from the stimulus sound to both cochleae. Recent studies within the past 10 years on sound localization and lateralization by BCDs, middle ear implants, and cartilage conduction hearing aids were discussed. Most studies showed benefits for sound localization or lateralization with bilateral devices. However, the judgment accuracy was generally lower than that for normal hearing, and the localization errors tended to be larger than for normal hearing. Moreover, it should be noted that the degree of accuracy in sound localization by bilateral BCDs varied considerably among patients. Further research on sound localization is necessary to analyze the complicated mechanism of bone conduction, including suprathreshold air conduction with bilateral devices.

## 1. Introduction

We are surrounded by many different sounds and we can easily know where they are and how far they are from us. This ability is called “localization”. According to Moore [1], the term “localization” refers to determining the direction and distance of a sound source. It is well known that sound localization in the horizontal plane is mediated by two cues: interaural time difference (ITD) and interaural level difference (ILD). The ITD is defined as the difference in arrival time between the two ears and is the most important cue to sound localization for low-frequency components [2]. The ILD is defined as the difference in the level of a sound at the two ears caused mainly by the head “shadowing” effect for high-frequency components [2]. Sound localization in the vertical plane is accomplished through filtering by the pinnae and the head itself. This filtering can be expressed in “head-related transfer functions (HRTFs)” [1]. The HRTF changes in the vertical and horizontal planes depending on the angle of incidence of the sound. So, with regard to hearing aids, there are differences in the HRTF depending on the angle at which the sound is presented from the loudspeaker when the device is worn, or where the device microphone is placed on the head. Related to sound localization, the term “lateralization” is used to describe the apparent location of the sound source within the head, when the stimulus is presented via headphones or bone vibrators. Sometimes the term “lateralization” is also used to judge whether the sound appears from the right or the left when presented by a loudspeaker [3].

Hearing loss affects sound localization and causes serious problems in daily life for the hearing-impaired. Häusler et al. (1983) [4] investigated the localization ability of persons with different types of hearing loss, such as conductive hearing loss (CHL), bilateral or unilateral sensorineural hearing losses, unilateral dead ear, and central hearing loss. For example, the localization ability in CHL is close to normal hearing if the loss does not exceed 25 dB HL. However, both unilateral and bilateral hearing losses greater than 35 dB HL affect the localization ability of both horizontal and vertical angle discrimination. Kramer et al. (1995, 1998) [5,6] investigated the extent to which individuals see themselves as being handicapped by gathering self-reports of 239 hearing-impaired persons with varying types of hearing loss. They showed that problems with sound intelligibility under noise and, indeed, auditory localization were considered as the most frequent disabilities.

The usefulness of bone conduction devices (BCDs) to assist persons with CHL, such as bone conduction hearing aids (BCHAs), was already pointed out in the early 1950s [7]. For a long time, unilateral fitting of BCHAs was commonly applied, even for persons with bilateral CHL caused by microtia, aural atresia, and chronic otitis media. One reason for the unilateral application is that the transcranial attenuation (TA) of bone conduction (BC) sound by a BCD is very small (10 dB), so it will stimulate both cochleae to almost the same extent [8]. In 1977, a percutaneous bone-anchored hearing aid (BAHA) was developed that avoids most of the drawbacks of conventional BCHAs [9,10]. Snik et al. (1998) [8] reported that sound localization, as indicated by the percentage of correct identification (within 45°), improved by 53% with binaural listening for three patients with BAHA(s) that were unilaterally or bilaterally fitted. Following this, significant improvement in sound localization with bilateral BAHAs has further been reported by Bosman et al. (2001) [3] and Priwin et al. (2004) [11]. In a systematic review of the literature from 1977 to 2011 by Janssen et al. (2012) [12], comparisons were made between unilateral and bilateral BCD(s) in participants with bilateral CHL or mixed hearing loss. The authors stated that the bilateral BAHA condition was shown to improve localization and lateralization, although it was difficult to appreciate the magnitude of this effect, given that only Priwin et al. (2007) [13] compared performances between hearing-impaired persons and a normal-hearing control group.

For bone-conducted sound lateralization, Kaga et al. (2001) [14] found, using a self-recording apparatus that measured ITD and ILD, that the abilities were maintained in many patients with bilateral microtia and aural atresia. Schmerber et al. (2005) [15] obtained time-intensity trading functions using ITD and ILD in the same ear from patients with bilateral congenital aural atresia, and showed that time-intensity trading was present in the patients. They concluded that a binaural fitting of BCHAs might optimize binaural hearing and improve sound lateralization, and recommended systematic bilateral fitting in aural atresia patients.

Further advances in technology have led to the development of various kinds of BCDs apart from conventional BCHAs with a steel-spring headband or with framed glasses. Reinfeldt et al. (2015) [16] categorized these as conventional skin-drive BCDs, passive transcutaneous skin-drive BCDs, percutaneous direct-drive BCDs, and active transcutaneous direct-drive BCDs. Recently, a non-surgical adhesive BCD has been made commercially available as well [17]. Moreover, cartilage conduction hearing aids (CCHAs) have been developed by Hosoi et al. (2010) [18], without the strong pressure of the steel spring as used in conventional BCHAs or surgical operations for BAHAs.

So far, research on sound localization thus has been carried out using the various kinds of devices mentioned above. Most of the studies have reported that bilaterally fitted devices showed more improved sound localization than the unilaterally fitted ones. As the basis, Zeitooni et al. (2016) [19] investigated the effects of binaural hearing with bilateral BCHAs, measuring the spatial release from masking, the binaural intelligibility level difference, the binaural masking level difference, and the precedence effect in adults with normal hearing. In all tests, the results with bilateral BC stimulation at the BCHA position illustrated an ability to extract binaural cues similar to BC stimulation at the mastoid position. They, however, did not test sound localization, the accuracy of which can be affected by various factors, such as the type of device, the participants, and the experimental method.

The present review aimed to discuss the factors affecting sound localization or lateralization, as well as their accuracy, for persons with bilateral (simulated) CHL using bilateral devices. For the first aim, the factors affecting sound localization and lateralization were classified, and the relevant research is discussed. For the second aim, regarding the accuracy of sound localization and lateralization using a multi-loudspeaker system, rather than a questionnaire such as “The Speech, Spatial and Qualities of Hearing Scale (SSQ) [20], the clinical literature related to persons with hearing loss or normal hearing was searched on “Google Scholar”. The keywords for this search were “bone conduction”, “localization”, “bilateral”, and “conductive hearing loss” for sound localization, and “bone conduction”, “lateralization”, “bilateral”, and “conductive hearing loss” for sound lateralization. The search was performed for literature from 2012 to August 2021 because Janssen et al. (2012) [12] had already reviewed the literature from 1977 to 2011. The strategy used to select the literature for the second aim was as follows.

**Figure 1:**
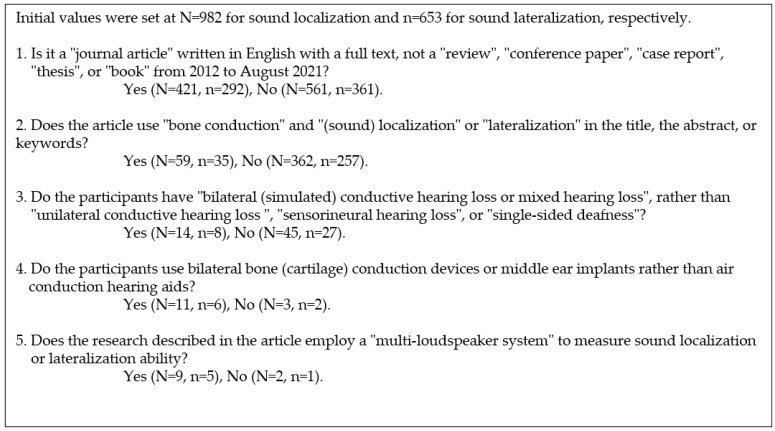
Screening process and the number of resulting hits regarding the literature about the accuracy of sound localization and lateralization using a multi-loudspeaker system (2012 to August 2021). “N=” and “n=” represent the hit number for “localization” and “lateralization”, respectively.

First, the keyword search conditions in “Google Scholar” were set to exclude “Include patents” and “Include quotes”. The search resulted in 1079 hits for sound localization and 670 hits for sound lateralization. These contents were sorted in descending order of relevance. The 1000 hits of the upper limit displayed from the top for sound localization and the 670 hits for sound lateralization were investigated. After inspection of the URLs, it was found that 982 hits for sound localization and 653 hits for sound lateralization were valid for analysis, after excluding links that displayed a “Not found” or “Not connected” message. These hit numbers were set as the initial values for screening for this review. The screening process and the number of resulting hits are shown in Figure 1. As a result, nine scientific articles for the review of sound localization and five for sound lateralization were picked up. The five articles for sound lateralization, however, were the same as the nine articles for localization, so these nine articles were selected. Furthermore, the nine articles for sound localization were categorized into those with normal-hearing participants with bilateral simulated CHL (three articles: Table 1) and those with bilateral CHL (six articles: Table 2).

## 2. Factors Affecting the Accuracy of Sound Localization or Lateralization

**Figure 2:**
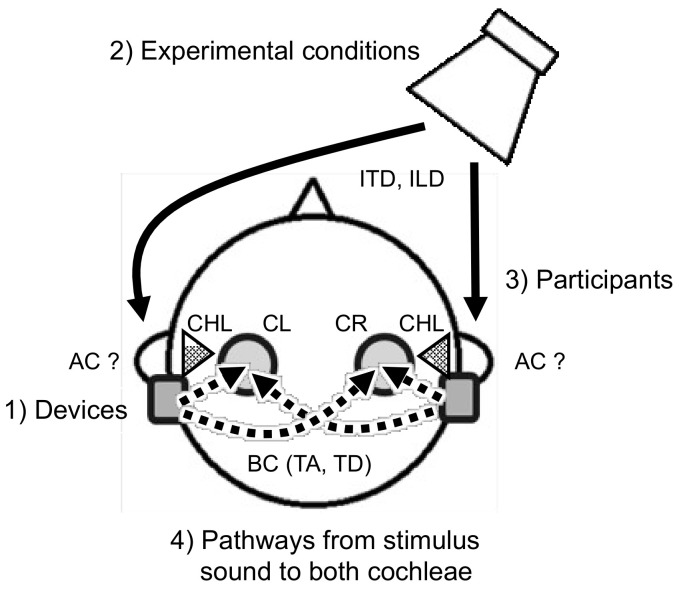
Factors affecting the accuracy of sound localization or lateralization of bone-conducted sound for a participant with (simulated) bilateral conductive hearing loss. Abbreviations: AC, air conduction; BC, bone conduction; CHL, conductive hearing loss; CL, left cochlea; CR, right cochlea; ITD, interaural time difference; ILD, interaural level difference; TA, transcranial attenuation; TD, transcranial delay.

Figure 2 schematically shows the factors that affect the accuracy of sound localization or lateralization of bone-conducted sound for a patient with bilateral CHL. These factors were roughly classified into four categories: (1) devices, (2) experimental conditions, (3) participants, and (4) pathways from the stimulus sound to both cochleae. Table 1 and Table 2 show the studies on sound localization or lateralization with details about the authors (year), devices, stimuli, presentation levels, setup, and test conditions for normal-hearing participants with simulated CHL and participants with bilateral CHL, respectively, in chronological order.

### 2.1. Devices

A conventional skin-drive BCHA that vibrates the bone via the skin consists of a microphone, an amplifier, and a BC transducer (vibrator or actuator) with a soft headband (softband), a steel spring headband, or spectacles [16]. The conventional electromagnetic bone transducers have a frequency curve that falls off below 0.25 kHz and above 4 kHz, with a resonance peak at 0.2–0.6 kHz [30]. This limited frequency range may affect the accuracy of front–back sound localization, because front–back localization appears to be coded in the energy contained within the 3–7 kHz bandwidth of a signal [31]. A giant magnetostrictive BC transducer with a frequency range from 0.5–30 kHz developed by Sakai et al. (2016) [30] may be useful for sound localization experiments. In addition, for bilaterally fitted BCDs, both the microphones and transducers are located a few centimeters apart in order to reduce acoustic feedback. Thus, the ITD and ILD are slightly affected depending on the position of the BCD’s microphones.

The principles and characteristics of the BCDs shown in Table 1 and Table 2 are, in brief, the following. Gawlizek et al. (2018a) [21] studied sound localization using two kinds of passive transcutaneous skin-drive BCDs. One BCD was the ADHEAR device (MED-EL, Innsbruck, Austria), where the transducer is glue-attached without pressure using an acrylic plate. The ADHEARs were also applied bilaterally by Ren et al. (2021) [28]. The audio frequency range is from 0.25 kHz to 8 kHz and signal processing occurs with automatic adaptive directional microphones (DMs) [32]. The other BCD is the Baha5 with a softband (Cochlear Inc., Mölnlycke, Sweden), which has an almost similar audio frequency range (Baha Connect: 0.25–7 kHz; Baha Attract: 0.25–6.3 kHz) [33]. Moreover, this device has a function of “Active Balanced Directionality”, controlled by the Scene Classifier, which gives wearers the ability to seamlessly blend between omnidirectional and directional settings [34]. The Bonebridge (BB) (MED-EL, Innsbruck, Austria), an active transcutaneous device, is a semi-implantable system consisting of an implantable part and an externally worn audio processor. The audio frequency range is from 0.25 kHz to 8 kHz and signal processing occurs with DM [35]. In CCHAs (HB-J1CC, Rion, Tokyo, Japan), the sound is delivered to the aural cartilage using a small and lightweight transducer that is attached to the aural cartilage without high-contact pressure. In contrast to the conventional BCHA, the transducer is placed without a headband, which is more comfortable for wearing, with better aesthetics. Surgery is not required with CCHAs, unlike with BAHAs and other middle ear implants [27]. The acoustical output of the transducer measured with the artificial mastoid has a mountain-shaped frequency response with a peak at 0.8–2 kHz [18]. The sound pressure levels in the external canal show double peaks at approximately 0.8 kHz and 2.5 kHz [36]. Nishimura et al. (2020) [27] used the device to study sound localization for the first time.

In studies on sound localization, various kinds of devices have been used for (simulated) CHL. The devices differ in microphone type and position, transducer positions, and signal processing. These differences may affect the accuracy of sound localization. Denk et al. (2019) [37] investigated the impacts of the microphone’s location, the signal bandwidth, and different equalization approaches, and showed that the microphone’s location was the governing factor for localization abilities with linear hearing devices. Regarding the relationship between adaptive DMs and localization in hearing aids, both the studies by Keidser et al. (2006) [38] and Van den Bogaert et al. (2006) [39] showed that independently operating adaptive DMs have an adverse effect on scores in a laboratory experiment. For synchronized adaptive DMs, the results are inconsistent. Namely, Keidser et al. (2006) [38] reported no benefit, while Ibrahim et al. (2013) [40] showed improvements for some stimuli. Johnson et al. (2017) [41] described that the difference between premium-feature hearing aids (i.e., with multi-channel adaptive DMs, pinna effect simulation, and an advanced synchronization function) and basic-feature hearing aids (i.e., with single-channel adaptive DMs and a basic synchronization function) was not significant in self-reported everyday sound localization. Caspers et al. (2021) [29] switched off adaptive DM and noise reduction in the setting of BCDs to avoid deterioration in localization performance.

Regarding the stimulation position of the bone-conducted sound, Stenfelt (2012) [42] reported that the median transcranial attenuation (TA) is 2 to 3 dB lower than at the mastoid when measured at the BCHA position. Dobrev et al. (2016) [43] investigated the influence of stimulus position on BC hearing sensitivity with a BC transducer attached using a headband. They concluded that stimulation on a position superior-anterior to the pinna provides more efficient BC transmission than stimulation on the mastoid. Moreover, the contact condition of the actuator at the stimulation position affects sound localization. Asakura et al. (2019) [44] reported that bone-conducted binaural sound localization performance could increase, depending on the contact force and the position of the actuator device.

### 2.2. Experimental Conditions

#### 2.2.1. Measurement Methods

When sound is presented by a loudspeaker in a sound field, two methods can be mainly used to measure the ability of sound localization. One is to identify one loudspeaker’s direction from multiple loudspeakers arranged in a semicircular or circular way relative to the participant. When multiple loudspeakers are arranged in a circle (e.g., see No. 1 and No. 2 in Table 1 and No. 4 in Table 2), it is easy to create front/back confusions, in that a stimulus in front of the participant is localized to the rear or vice versa [45]. The frequency of front/back confusions tends to increase as the bandwidth of the stimulus is decreased [46]. Front/back confusion is caused by the difficulty of localization using the ITD and the ILD in the experimental room, although moving one’s head or experience from the surrounding sound environment can help to localize a sound source in daily life. The second method is to discriminate the minimum audible angle (MAA), which is defined as the smallest detectable difference between the azimuths of two identical sounds [47]. In this method, immediately after presenting the reference sound, the sound source’s position is shifted to the left or right, and the MAA is measured by asking the participant to answer whether the test sound is heard from the left or right of the reference. Already in 1958, Mills [47] described that the MAA for a tone of 1 kHz or higher is about 1 degree at an azimuth of 0 degrees. The discrimination task is also easy for children to measure the ultimate sensitivity of the localization system [4]. For example, Lovett et al. (2012) [48] reported that children showed adult levels of performance from age 3 years for left–right discrimination, and from age 6 years for localization. Asp et al. (2016) [49] developed a corneal reflection eye-tracking technique to record pupil positions toward spatially distributed continuous auditory and visual stimuli to assess horizontal sound localization accuracy from 6 months of age. They showed that the method provides an objective and fast assessment of horizontal sound localization accuracy.

#### 2.2.2. Stimulus Conditions

Pink noise, white noise, and broadband noise are often used as the stimulus sound, as shown in Table 1 and Table 2. When used with wideband frequency, it is necessary to confirm whether the stimulus sound is sufficiently reproduced in the device’s frequency range. Yost et al. (2014) [50] described that the accuracy of sound source localization increases as the bandwidth of the stimulus sound increases, and that stimuli with a wide range of one octave or more have the best sound source localization accuracy. The onset duration of the stimulus also affects sound source localization [51]. Stimulus levels are often used at a level of 65 dB SPL or 65 dB A, as shown in Table 1 and Table 2, which corresponds to the intensity level of conversation. When the stimulus presentation level becomes larger, it exceeds the earplugged air conduction threshold or the actual patient’s threshold with CHL. In this case, both the bone-conducted sound, via bilaterally fitted BCDs, and the suprathreshold air-conducted sound may be presented simultaneously to the cochleae and interfere with the sound localization cues (Figure 2).

### 2.3. Participants

There are merits and demerits in sound localization experiments for employing, respectively, participants with normal hearing (bilateral simulated CHL) and patients with bilateral CHL.

#### 2.3.1. Normal-Hearing Participants with Simulated CHL

Normal-hearing participants are used to simulate bilateral CHL by blocking their ears with earplugs (and earmuffs) or earmolds. Normal-hearing participants with simulated CHL have the advantages of normally developed hearing, and the BC thresholds of the left and right ears under masking can be accurately measured. The participants thus can be assumed to be a homogeneous study group. However, there is a limit of sound insulation by earplugs and earmuffs, and the audiogram of simulated CHL depends on the sound insulation performance. For example, in an experiment to investigate the effect of simulated unilateral hearing loss on horizontal sound localization by Asp et al. (2018) [52], the degrees of hearing loss were mild hearing loss (average threshold of 30 dB HL across 0.5, 1, 2, and 4 kHz) with an earplug, and moderate hearing loss (average threshold of 43 dB HL across 0.5, 1, 2, and 4 kHz) with an earplug and earmuff. Furthermore, both audiogram configurations showed larger hearing loss in the high frequencies than in the low frequencies. Compared with actual patients with CHL, these degrees of simulated hearing loss and the audiogram configuration may differ substantially.

#### 2.3.2. Patients with Bilateral CHL

For patients with bilateral CHL, it is clinically meaningful to examine their sound localization ability. The heterogeneity of the study group with respect to the duration of deafness, the degree of hearing loss, the symmetry of hearing, and the period of device use makes it difficult to generalize the results. Furthermore, there are few reports on how localization accuracy is affected by whether the CHL is congenital or acquired. In the case of congenital aural atresia and microtia, the auditory system may not always be fully developed for both ears. Kaga et al. (2016) [53] carried out a sound lateralization test (ILD and ITD) in 18 patients with unilateral microtia and atresia, after reconstruction of the auricle and external canal and fitting a canal-type hearing aid for the operated ear. Their results showed that the ability to discriminate the ILD was acquired in all of the patients, whereas that to discriminate ITD was acquired in only half of the patients. They stated that the difference must be caused by late-development brain plasticity for binaural hearing. Caspers et al. (2021) [29] reported that bilaterally fitted patients with bilaterally acquired hearing loss, as well as patients with congenital hearing loss, were capable of localizing sounds (quite) accurately. For the obtained bilateral BC thresholds, they described that sound lateralization was more accurate in patients with symmetric and near-normal BC thresholds when compared with patients with either asymmetric BC thresholds or patients with BC thresholds of 25 dB and higher, and that normal symmetric thresholds did not warrant good localization. Here, when the degree of CHL in both ears became larger in a patient with bilateral CHL, it was difficult to obtain an actual BC threshold due to over-masking (the so-called “masking dilemma”) [54].

When the participants are children, their ages can affect the ability of sound localization. From measurement of ITD and ILD with a self-recording apparatus, Kaga (1992) [55] showed that the ability to localize sound sources rapidly developed between the ages of 5 and 6 years. In addition, for children with bilateral congenital microtia, Ren et al. (2021) [28] reported that the improvement in sound localization was also negatively related to the malformation degree of the patient’s head. Apart from this, the ability of sound localization can improve with training. Following tests with 11 participants with unilateral severe to profound hearing loss, Firszt et al. (2015) [56] reported that the eight participants with the poorest localization ability improved significantly following training, while the three participants with the best pre-training ability showed the least training benefit. Taking all the abovementioned factors into consideration, in experiments with patients, it is generally difficult to have a group with the same patient background.

### 2.4. Pathways from the Sound Source to the Cochleae

Sound localization by binaural hearing with devices is mainly mediated by two pathways: (1) the pathways from the sound source to the microphones of the bilateral devices, and (2) the pathways from the bone-conducted sound induced by both devices to both cochleae (Figure 2).

#### 2.4.1. Pathways from the Sound Source to the Microphones of the Bilateral Devices

The ITD detection threshold varies depending on the type of sound (e.g., the use of a band-limited random noise from 0.15–1.7 kHz, a 1 kHz tone, or a 1-millisecond click) and ranges from 9 to 28 μs [57]. ITD reaches its maximum when the sound arrives from the side, and its value is then about 650 μs [2]. The detection threshold of ILD is about 1 to 2 dB [2].

#### 2.4.2. Pathways from Bone-Conducted Sound induced by Devices to the Cochleae

It is generally accepted that bone-conducted sound transmission in the human skull is linear, at least for frequencies between 0.1 and 10 kHz and up to 77 dB HL [58]. However, the relationship between the mechanism of bone-conducted sound propagation within the skull and BC hearing has not yet been fully elucidated. Eeg-Olofsson (2012) [58] reported that the main components that contribute to BC hearing are: the occlusion effect, middle ear ossicle inertia, inner ear fluid inertia, compression and expansion of the cochlea, and the cerebrospinal fluid pathway. When both devices stimulate the left and right cochleae, an ILD by the TA and an ITD by the transcranial delay (TD) between the ipsilateral and the contralateral cochleae to the stimulation may assist sound localization.

- Transcranial attenuation (TA):

Stenfelt et al. (2012) [42] studied TA in 28 cases of unilateral deafness using four stimulus positions (ipsilateral, contralateral mastoid, ipsilateral, and contralateral position) for a BCHA at 31 frequencies from 0.25 to 8 kHz. The results showed that with stimulation at the mastoid, the median TA was 3 dB to 5 dB at frequencies up to 0.5 kHz and close to 0 dB between 0.5 to 1.8 kHz. The TA was close to 10 dB at 3 to 5 kHz, and became slightly less at the highest frequencies measured (4 dB at 8 kHz). Furthermore, the intersubjective variability was large for each frequency (around 40 dB), but there were small differences in the general trends of TA between individuals. For normal-hearing participants, Stenfelt et al. (2013) [59] reported that the TA showed almost the same tendencies as in participants with unilateral deafness. Recently, Röösli et al. (2021) [60] reported that TA is affected by stimulus location, the coupling of the bone conduction hearing aid to the underlying tissue, and the properties of the head (such as the geometry of the head, thickness of the skin and/or skull, changes due to aging, iatrogenic changes such as bone removal during mastoidectomy, and occlusion of the external auditory canal).

- Transcranial delay (TD):

TD between the ipsilateral and contralateral cochleae with stimulation by a BCD on one side is related to the propagation velocity of bone-conducted sound in the skull. Franke (1956) [61] placed two pickups on the frontal and parietal regions of a human skull and observed the BC velocity as the difference in the waveform between the two pickups when stimulating the forehead. As a result, the propagation velocity increased from low frequencies to high frequencies: it was about 150 m/s near frequencies of 0.5 kHz and about 300 m/s at frequencies above 1.5 kHz, which then almost remained constant. Wigand et al. (1964) [62], however, reported that the BC velocity of the skull base is 3000 m/s. Contrary to this, by using a psychophysical method, Tonndorf et al. (1981) [63] measured the propagation velocity of bone-conducted sound and reported that indeed it was about 55 m/s near frequencies of 0.5–0.75 kHz and about 330 m/s at frequencies above 2 kHz for the human skull. By measuring the mechanical point impedance from 27 positions on the skull surface in six intact cadaver heads, Stenfelt and Goode (2005) [64] reported that the phase velocity in the cranial bone is estimated to increase from around 250 m/s at 2 kHz to 300 m/s at 10 kHz. Although the propagation velocity value in the skull thus differs depending on the frequency of the bone-conducted sound, the object (dry skull, living subject, human cadaver), and the measurement method, this velocity indicates the TD of the bone-conducted sound for ipsilateral mastoid stimulation between the ipsilateral and the contralateral cochleae. Zeitooni et al. (2016) [19] described that the TD between the cochleae for mastoid placement of BC stimulation is estimated to be 0.3 to 0.5 ms at frequencies above 1 kHz, while there are no reliable estimates at lower frequencies.

As described above, the bone-conducted sound induced via bilateral devices can cause complicated interference for the bilateral cochleae due to TA and TD. Farrel et al. (2017) [65] measured ITD and ILD from the intracochlear pressures and stapes velocity conveyed by bilateral BC systems. They showed that the variation of the ITDs and ILDs conveyed by bone-anchored hearing devices systematically modulated cochlear inputs. They concluded that binaural disparities potentiate binaural benefit, providing a basis for improved sound localization. At the same time, transcranial cross-talk could lead to complex interactions that depend on cue type and stimulus frequency.

## 3. Accuracy of Sound Localization and Lateralization Using Device(s)

As mentioned above, previous studies have shown that sound localization by bone-conducted sound with bilaterally fitted devices involves a greater variety of factors than sound localization by air-conducted sound. Next, a review was made to assess how much the accuracy of sound localization by bilaterally fitted devices differs from that with unilaterally fitted devices or unaided conditions for participants with bilateral (simulated) CHL and with normal hearing. The methodology of the studies is shown in Table 1 and Table 2.

### 3.1. Normal-Hearing Participants with Simulated CHL

Gawliczek et al. (2018a) [21] evaluated sound localization ability using two non-invasive BCDs (BCD1: ADHEAR; BCD2: Baha5 with softband) for unilateral and bilateral simulated CHL with earplugs. The mean absolute localization error (MAE) in the bilateral fitting condition improved by 34.2° for BCD1 and by 27.9° for BCD2 as compared with the unilateral fitting condition, thus resulting in a slight difference of about 7° between BCD1 and BCD2. The authors stated that the difference was caused by the ILD and ITD from different microphone positions between the BCDs. Gawliczek et al. (2018b) [22] further measured the audiological benefit of the Baha SoundArc and compared it with the known softband options. No statistically significant difference was found between the SoundArc and the softband options in any of the tests (soundfield thresholds, speech understanding in quiet and in noise, and sound localization). Using two sound processors rather than one improved the sound localization error by 5°, from 23° to 28°.

Snapp et al. (2020) [23] investigated the unilaterally and bilaterally aided benefits of aBCDs (ADHER) in normal-hearing listeners under simulated (plugged) unilateral and bilateral CHL conditions using measures of sound localization. In the listening conditions with bilateral plugs and bilateral aBCD, listeners could localize the stimuli with a high degree of accuracy. The response gains reached that of normal hearing performance for all levels, although the target response plots indicated a larger scatter and a worse MAE than in normal hearing conditions. The results for the unilateral application of the aBCD condition with bilateral plugs, however, showed a clear localization bias towards the aBCD side.

### 3.2. Patients with Bilateral CHL

Fan et al. (2020) [25] compared the effects of one BCD (BB) and bilateral BCDs (BB plus contralateral ADHEAR) on sound localization abilities in patients with bilateral microtia–atresia. The results showed that the response accuracy was significantly better with bilateral BCDs (22%) than with unilateral BCDs (16%). However, the percentage with bilateral BCDs did not reach the level of the unaided condition. The bias angles following unilateral and bilateral BCDs were 34.1° and 26.4°, respectively, indicating ipsilateral bias directed to the side of BB implantation. The authors stated that these findings may be explained by the partial re-establishment of ITDs and ILDs by bilateral BCDs. With regard to this partial re-establishment, they considered that the BB might have provided a relatively stronger stimulation of both cochleae compared with the contralateral ADHEAR. Ren et al. (2021) [28] also used ADHEARs bilaterally for 12 children with mild to severe bilateral CHL due to congenital microtia. They stated that unilateral fitting of ADHEAR did not improve the sound localization ability, while bilateral fitting demonstrated instant improvement in half of the patients, in that the root mean square error (RMSE) decreased from 67.9 ± 10.9° (unaided condition) to 33.7 ± 4.9° (bilateral fitting). For the other half of the patients, however, no significant difference was found in the RMSE between the unaided condition of 49.7 ± 15.0° and the bilateral fitting of 57.7 ± 15.1°. Thus, they showed that the improvement in sound localization ability under bilateral fitting strongly correlated with the unaided sound localization ability: patients who perform worse when unaided tend to benefit more.

Caspers et al. (2021) [29] investigated sound localization in 15 patients bilaterally fitted with BCDs (Baha4 or Baha5) and explored clinical methods to improve localization accuracy. Sound localization was measured at baseline, and settings to optimize sound localization were added to the BCDs. At 1 month, sound localization was assessed again and localization was practiced with a series of sounds with visual feedback. At 3 months, localization performance, device use, and questionnaire scores were determined again. As a result, at baseline, one patient with congenital hearing loss demonstrated near excellent localization performance, and four other patients (three with congenital hearing loss) localized sounds (quite) accurately. Seven patients with acquired hearing loss were able to lateralize sounds (i.e., identify whether the sounds were coming from the left or right side) but could not localize sounds accurately. Three patients (one with congenital hearing loss), however, could not lateralize sounds correctly. Nevertheless, the authors concluded that the majority of experienced bilateral BCD users could lateralize sounds and one-third were able to localize sounds (quite) accurately, with robust performance over time.

Dun et al. (2013) [24] investigated whether children with bilateral CHL benefitted from their second device (i.e., the bilateral BCD (Baha)). Spatial resolution was tested with MAA in the bilateral and monaural listening conditions. The MAA decreased from 57° in the best monaural condition to 13° in the bilateral condition, thus demonstrating the advantage of bilateral BCD fitting in children with bilateral CHL. In a related study, Besten et al. (2020) [26] characterized the lateralization (MAA) of sounds and localization of sounds in children with bilateral CHL when listening with either one or two percutaneous BCDs (Baha Divino, Baha BP100, or Baha4). For lateralization of sound, in seven out of the 10 children, the MAA was 90° in one or both of the unilateral conditions, and equal to or less than 15° in the bilateral condition. The result of lateralization in the bilateral BCD condition was close to normal in nearly all the children. Sound localization thus was better with bilateral BCDs than in the unilaterally aided conditions. However, most children showed a bimodal response pattern, reflecting sound lateralization and not sound localization in the sound localization test.

Nishimura et al. (2020) [27] evaluated sound localization for patients with bilateral aural atresia using CCHAs in three conditions: unaided, aided with previously used hearing aids (air conduction HAs or BCHAs), and aided with CCHAs. The ability to distinguish sounds originating from left or right for participants aided with CCHAs was significantly better than that for other conditions. Compared with that in patients aided with previously used aids, no difference in front-back misidentification was found. The percent correct rates of 0.88 for left and 0.9 for right sound localization by CCHAs showed statistically significant improvements compared with the percent correct rates of 0.77 (left) and 0.64 (right) obtained with previously used HAs. The authors hypothesized that the reason might be the contribution of vibration sensation due to lower contact pressure by the transducer of CCHAs in comparison with conventional BCHAs.

## 4. Conclusions

As reviewed above, most of the recent studies on sound localization and lateralization have shown that performance with bilaterally fitted devices was better than that with unilaterally fitted device for bilateral (simulated) CHL. However, the judgment accuracy was generally lower than that for normal hearing, and the localization errors tended to be larger than for normal hearing. It should also be noted that the degree of accuracy in sound localization with bilateral BCDs varied considerably among patients. Many factors such as the type of device, the experimental conditions, participants, and pathways from the stimulus sound to both cochleae can affect the results. Especially, it is unclear whether localization with bilaterally fitted devices, for which the presentation level exceeds the threshold in bilateral (simulated) conductive hearing loss, involves both air-conducted sound and bone-conducted sound. Further research on sound localization is necessary to analyze the complicated mechanism of BC, including suprathreshold air conduction with bilateral devices.

## Figures and Tables

**Table 1:** Studies on sound localization or lateralization with bone conduction for simulated conductive hearing loss from 2012 to August 2021.

No.	Authors (Year)	Participants	Devices	Stimuli	Presentation Levels	Setup	Test Conditions
1	Gawliczek et al. (2018a) [21]	15 participants with induced bilateral CHL	aBCD(s) (ADHEAR) and BCD(s) attached to a softband (Baha5)	White noise (200 ms duration)	Between 60 and 65 dB SPL	Twelve loudspeakers (aligned in a horizontal circular set up: an angular interval of 30°)	Evaluation of BCD1 (ADHEAR), comparison between unilateral and bilateral fitting, and comparison between BCD1 and BCD2 (Baha 5)
2	Gawliczek et al. (2018b) [22]	15 participants with simulated bilateral CHL with a combination of ear plugs and silicon mould material	BCD(s) (Baha5) (SoundArc and a softband)	White noise (200 ms duration)	60, 65, and 70 dB SPL	Twelve loudspeakers (aligned in a horizontal circular set up: an angular interval of 30°)	(i) Unaided, (ii) aided with one BAHS sound processor mounted on a SoundArc, (iii) aided with 2 BAHS sound processors on a single SoundArc, (iv) aided with one BAHS sound processor on a softband, and (v) aided with 2 BAHS sound processors on a single soft band
3	Snapp et al. (2020) [23]	11 listeners with simulated CHL with plug(s)	aBCD(s) (ADHER)	Broadband (BB; 0.5–20 kHz), high-pass (HP; 3–20 kHz), and low-pass (LP; 0.5–1.5 kHz) noise bursts (150 ms duration)	45, 55, and 65 dB A	Twenty-four loudspeakers (the horizontal (±70°) and vertical (+40°/−30°) planes (The speakers were covered by a black, sound emitting curtain)	Normal hearing, unilateral plug, unilateral plug + ipsilateral aBCD, unilateral plug +ipsilateral aBCD+ contralateral pinna mold, bilateral plugs, bilateral plugs + bilateral aBCDs, and bilateral plugs + unilateral aBCD

Abbreviations: aBCD, adhesive bone conduction device; BAHS, bone-anchored hearing systems; CHL, conductive hearing loss.

**Table 2:** Studies on sound localization or lateralization with bone conduction device(s) for bilateral conductive hearing loss from 2012 to August 2021.

No.	Authors (Year)	Participants	Devices	Stimuli	Presentation Levels	Setup	Test Conditions
1	Dun et al. (2013) [24]	10 children with severe bilateral CHL due to congenital major or minor ear anomalies with microtia or anotia or due to resistant chronic inflammation	Unilateral (right or left) BCD and bilateral BCDs (Baha)	Broadband noise; (0.5 to 20 kHz, 500 ms duration)	Randomly in 10 dB steps within the 40 to 70 dB SPL range	MAA: two loudspeakers (±90°, ±60°, ±30°, ±15°, ±10°, and ±5°)	Unilateral condition (right or left) and bilateral condition
2	Fan et al. (2020) [25]	32 patients with congenital bilateral microtia-atresia	Bilateral BCDs (BB and l ADHEAR)	White noise	65, 70, and 75 dB SPL	Seven loudspeakers (30° interval from −90° to +90°)	Three conditions: unaided, unilateral BCD (BB) and bilateral BCDs (BB plus contralateral ADHEAR)
3	Besten et al. (2020) [26]	10 children with congenital bilateral CHL and 1 child with acquired bilateral CHL	One or two percutaneous BCDs (Baha Divino, Baha BP100, or Baha4)	MAA: a broadband noise burst (bandwidth 0.5–20 kHz, 500 ms duration) Sound localization: broadband noise bursts (bandwidth 0.5–8 kHz, 150 ms duration)	MAA: 55, 60 or 65 dB SPL Sound localization: 50, 60, or 70 dB SPL	MAA: two loudspeakers (±90°, ±60°, ±30°, ±15°, ±10°, and ±5°) Sound localization: −75° (left) to +75° (right).	Unilateral BCD on the left side, unilateral BCD on the right side, and bilateral BCDs
4	Nishimura et al. (2020) [27]	13 patients with bilateral aural atresia	CC hearing aids	Pink noise (500 ms duration including a rise/fall time of 50 ms)	65 dB SPL	Eight loudspeakers (full circle at 45° interval)	Unaided, aided with previously used hearing aids (air conduction or BC hearing aids), and aided with CC hearing aids
5	Ren et al. (2021) [28]	12 children with mild-to-severe bilateral conductive hearing loss due to congenital microtia	Unilateral or Bilateral BCD(s) (ADHEAR)	A recorded gunshot sound	65 dB SPL	Twelve loudspeakers (a semi-circle, ranging from −82.5° to 82.5° with 15° intervals	Unaided, unilateral aided and bilateral aided
6	Caspers et al. (2021) [29]	15 adults with bilateral CHL and mixed HL (congenital and acquired HL)	Percutaneous BCDs (Baha4 or Baha5)	Broadband noise (BB; 0.5 to 20 kHz), high-pass (HP; 3–20 kHz) and low-pass (LP; 0.5–1.5 kHz)	45, 55, and 65 dB SPL for BB stimuli, and 55 dB SPL for HL and LP stimuli	Twenty-four loudspeakers positioned on an arc between +70° (right) and −70° (left) azimuth and between +40° (up) and −30° (down)	Unilateral aided conditions (right and left) and bilateral aided condition

Abbreviations: BCD, bone conduction device; BB, bonebridge; CHL, conductive hearing loss; CC, cartilage conduction; HL, hearing loss; MAA, minimum audible angle.

## Data Availability

Not applicable.

## Abbreviations

aBCD	adhesive bone conduction device
BAHA	bone-anchored hearing aid
BB	Bonebridge
BC	bone conduction
BCDs	bone conduction devices
BCHAs	bone conduction hearing aids
CCHAs	cartilage conduction hearing aids
CHL	conductive hearing loss
DM	directional microphone
HL	hearing level
HRTF	head-related transfer function
ILD	interaural level difference
ITD	interaural time difference
MAA	minimum audible angle
MAE	mean absolute localization error
μs	microsecond
RMSE	root mean square error
TA	transcranial attenuation
TD	transcranial delay
